# Effects of Organic Xenobiotics on *Tenebrio molitor* Larvae and Their Parasite *Gregarina polymorpha*

**DOI:** 10.3390/biology13070513

**Published:** 2024-07-10

**Authors:** Viktoriia Lazurska, Viktor Brygadyrenko

**Affiliations:** 1 Department of Medical Biology, Pharmacognosy, Botany and Histology, Dnipro State Medical University, Vernadsky St. 9, 49044 Dnipro, Ukraine; lazurskaviktoriia@gmail.com; 2 Department of Zoology and Ecology, Faculty of Biology and Ecology, Oles Honchar Dnipro National University, Gagarin Av. 72, 49010 Dnipro, Ukraine; 3 Department of Anatomy, Histology and Pathomorphology of Animals, Faculty of Veterinary Medicine, Dnipro State Agrarian and Economic University, Sergiy Efremov St. 25, 49600 Dnipro, Ukraine

**Keywords:** Eugregarinorida, pollutants, xenobiotic, changes in body mass, parasitic system

## Abstract

**Simple Summary:**

Substances that are absent in the biosphere, but have been synthesized by humans in recent decades, are called xenobiotics. They are released into the environment in large volumes and can actively affect invertebrates and their intestinal parasites or commensals. Such an influence of xenobiotics on parasite–host relationships remained poorly studied until recently. In this article, the host–parasite system “larva of *Tenebrio molitor—Gregarina polymorpha*” under the influence of 24 organic compounds is investigated. We found that several substances increase the mortality of *T. molitor* larvae, and substances such as diphenyl ether, benzyl alcohol, catechol, and 3-aminobenzoic acid, on the contrary, reduce the number of parasites (*G. polymorpha*) without significantly affecting host insects (larva of *T. molitor*). Thus, various xenobiotics can destroy the parasite–host relationship, having a greater effect on the host in some cases and on the parasite in other cases.

**Abstract:**

Environmental contamination with xenobiotics affects organisms and the symbiotic relations between them. A convenient object to study relationships between parasites and their hosts is the host–parasite system “*Tenebrio molitor* Linnaeus, 1758 (Coleoptera, Tenebrionidae)*—Gregarina polymorpha* (Hammerschmidt, 1838) Stein, 1848 (Eugregarinorida, Gregarinidae)”. For this experiment, we took 390 *T. molitor* larvae and 24 organic compounds. Groups of mealworms, 15 in each, were subjected to those compounds for 10 days. Then, we recorded the vitality of both the larvae of *T. molitor* and *G. polymorpha*. To assess how *G. polymorpha* had affected the hosts’ wellbeing, we looked for changes in the larvae’s body mass and compared them to the number of gregarines in their intestines. The vitality of the larvae was inhibited by cyclopentanol and 2-naphthol. The intensity of gregarine invasion was reduced by diphenyl ether, benzyl alcohol, catechol, and 3-aminobenzoic acid. No effect on the number of gregarines was produced by 3,4,5-trihydroxybenzoic acid, cyclohexanemethanol, phenol, benzalkonium chloride, maleic anhydride, cyclohexanol, resorcin, benzoic acid, 2-methylfuran, terpinen-4-ol, 1-phenylethylamine, dibutyl phthalate, 3-furancarboxylic acid, 5-methyl furfural, 6-aminohexanoic acid, succinic anhydride, o-xylene, and benzaldehyde. In the infected *T. molitor* individuals, the mean number of *G. polymorpha* equaled 45 specimens per host. The groups of smaller mealworms had fewer gregarines. Positive correlation was seen between growth rates of *T. molitor* larvae and the intensity of invasion by gregarines.

## 1. Introduction

*Tenebrio molitor* Linnaeus, 1758 (Coleoptera, Tenebrionidae) is now widespread all around the globe [1]. Imagoes and larvae of this beetle live under the bark of trees, in soil, under foliage, and in other humid, dark places [2]. The yellow mealworm beetle is mostly active at night, feeding on animal and plant products [3,4]. At the same time, it is a storage pest of grain and grain products [5]. In case of mass reproduction, it is able to destroy up to 15% of storage reserves [6]. *Tenebrio molitor* feeds on grain, and often lives in it as well, laying eggs and polluting the supplies with feces [7].

Similarly to other insects with complete metamorphosis, *T. molitor* undergoes four stages of ontogenesis: egg, larva, pupa, and imago. Environmental factors such as temperature, humidity, and access to food play crucial role in how long any *T. molitor* specimen may spend in each ontogenesis stage [8]. *Tenebrio molitor* is able to effectively adapt to unfavorable conditions and survive in them. It does not require a reliable source of water, since it is able to obtain moisture from food or the atmosphere [9]. Because of its low demand and easy maintenance, *T. molitor* is an insect that is cultivated at the industrial scale [10] and is used in many spheres. For example, mealworms are reared as food for pets: they are added to the diets of many fishes, birds, and arthropods, and are used as bait in fishing [1]. Dried *T. molitor* have a high content of protein (~50%) and many microelements, and therefore are notable edible insects [11,12]. Mass animal husbandry affects the environment by forming large volumes of greenhouse gasses and ammonia and also uses considerable amounts of water, energy, and areas of land. Therefore, insects are now being considered an alternative source of nutrients [13]. In 2021, the European Union certified the powder of mealworms as a food raw material [14].

*Tenebrio molitor* is also used in research in biology, biochemistry, evolution, etc. [15,16,17]. Researchers found that because of their gut microflora, mealworms are able to consume and degrade polystirol, polypropylene, and polyvinyl chloride [18]. At the same time, 35% to 50% of polymer is converted into carbon dioxide, while the rest does not remain in the organism for long [19]. Thanks to this ability, *T. molitor* can potentially improve the ecological condition of the planet. *Tenebrio molitor* is also used in the production of biodiesel [20], as a source of chitin and chitosan [21], and as bioactive extracts and compounds [22,23]. Thus, *T. molitor* is significant for mankind and has potential in various other spheres [24].

Gregarines belong to the Apicomplexa phylum and parasitize most groups of invertebrates. They have been studied only fragmentarily [25]. Mealworms harbor gregarines such as *Gregarina cuneata* Stein, 1848; *G. polymorpha* (Hammerschmidt, 1838) Stein, 1848; and *G. steini* Berndt, 1902, whereas the imago has not been found to be infected by any of those, only by *G. niphandrodes*, which, in turn, does not parasitize the larva stage [26,27,28].

The life cycle of gregarines comprises sporogony and gametogony [29]. Gregarine infection starts with the consumption of invasive oocysts that are released to the environment in the feces of already infested organisms [30]. In the host, oocysts release sporozoites, which attach to the cells through mucrons or epimerites [31]. Gregarines live in the digestive tract or internal cavity of their hosts.

Over 1600 gregarines have been discovered so far, but the number is suspected to be much higher—over a million—due to their high specificity (the species of gregarines that associate with a host can change over its life cycle) and wide distribution [32,33]. The effect gregarines have on their hosts has been the subject of numerous experimental studies. Gregarines are often considered commensals, i.e., such that they obtain benefits from another organism but provide no benefits in return, but at the same time cause no harm [34,35,36]. Despite this fact, from time to time they demonstrate a unique action towards their hosts, being both harmful and beneficial. Research has demonstrated the various effects gregarines have on their hosts: they can arrest development, cause greater mortality, and inhibit reproduction [36,37]. Excessive infestations of gregarines can occupy entire intestines, obstructing the entrance for food, ruining the intestinal wall of their host, and provoking other complications [38]. Growth rates of body mass in the host are related to the composition of its diet, whereas invertebrates that feed on dozens of types of feeds are not necessarily associated with the composition of diet [39,40]. Gregarines cause harm to some adaptive properties of dragonflies by reducing fat in their bodies, thus negatively impacting their reproduction [41]. Gregarines were observed to hinder the development of larvae and pupae in the *Aedes triseriatus* (Say, 1823) mosquito (Diptera, Culicidae) [42]. However, in other cases, gregarine infestation could give their host advantages. For example, in conditions of food scarcity or starvation, they had a positive effect on their hosts, including *Enallagma boreale* (Selys, 1875) (Odonata, Coenagrionidae) [43] and gregarine infection boosted the rates of development of their host *Ctenocephalides felis* (Bouché, 1835) (Pulicidae, Ctenocephalides) [44].

Issues of contamination of the environment have become especially acute in recent decades [45]. Xenobiotics are determined as alien compounds in the biosphere [46] and comprise the main group of pollutants. Xenobiotics include colorings, pharmaceutical drugs, means of personal hygiene, and pesticides [47]. They are resilient to degradation, and therefore are able to accumulate in large concentrations in the soil, damaging the chemical stability of the soil environment [48]. They enter surface and ground water in urban areas via industrial and municipal discharges of waste water, and also agricultural waste. Organisms living in water bodies are also among those harmed [49,50]. Xenobiotics and their metabolites can affect various physiological changes in organisms, causing immunological and reproductive malfunctions and altered cardiovascular parameters or systems of organs [51]. These pollutants can remain in the fatty tissues for many years and cause chronic problems such as growth arrest, relapse of diseases, long brain malfunctions, and cancer [52]. Substantial accumulation of xenobiotics in the environment threatens the existence of entire ecosystems. By affecting the populations of certain species, xenobiotics also cause the destruction of symbiotic relationships in ecosystems. In laboratory conditions, it is possible to accurately monitor the effects of xenobiotics on unicellular parasites in the host’s body and how this will affect the parasite system in general. The substances studied in this article are widely used in everyday life, construction, and the chemical industry, and, therefore, every year they enter the environment and accumulate near places of waste generation and storage. This causes the accumulation of xenobiotics in the bodies of organisms that live on the surface and in the upper horizons of the soil and feed on plant and animal remains, i.e., they are saprophages. Larvae of *T. molitor* are often found in places of accumulation in household and other types of organic waste, where the probability of the accumulation of many types of xenobiotics is highest. That is why they are a convenient object for evaluating the impact on the host–parasite system.

The objective of this study was to assess the effects of 24 organic compounds on the host–parasite system “*Tenebrio molitor—Gregarina polymorpha*”.

## 2. Materials and Methods

For this experiment, we chose 24 organic compounds (Table 1) and took 390 larvae of *T. molitor* from one laboratory population, where, prior to the experiment, they had been kept in the same conditions of temperature, humidity, and light for 3 months. All the larvae had the same access to food and fed on one medium, and had the same chance of becoming infected by gregarines. During the experiment, groups of 15 mealworms (5 in one of three cups) were exposed to each compound. The compounds affected the development of the larvae through their respiratory [53] and digestive systems. The control group comprised 30 larvae.

Each larva was designated with a marker (one to five tergites were marked depending on assigned number) and transported in a 500 mL plastic cup with substrate of flaked oat groats. All the cups contained substrate from one lot of groats of the same density and water content. Inside the substrate (closer to the cup bottom), we dropped 0.05 mL of each studied compound using a pipette [54]. Onto the surface of the substrate, we put 5 g of inner leaf of cabbage to offer additional nutrients and provide optimal environmental humidity [55]. All the cups were on the surface of the table under direct sunlight in the conditions of the same humidity and temperature.

**Figure 1:**
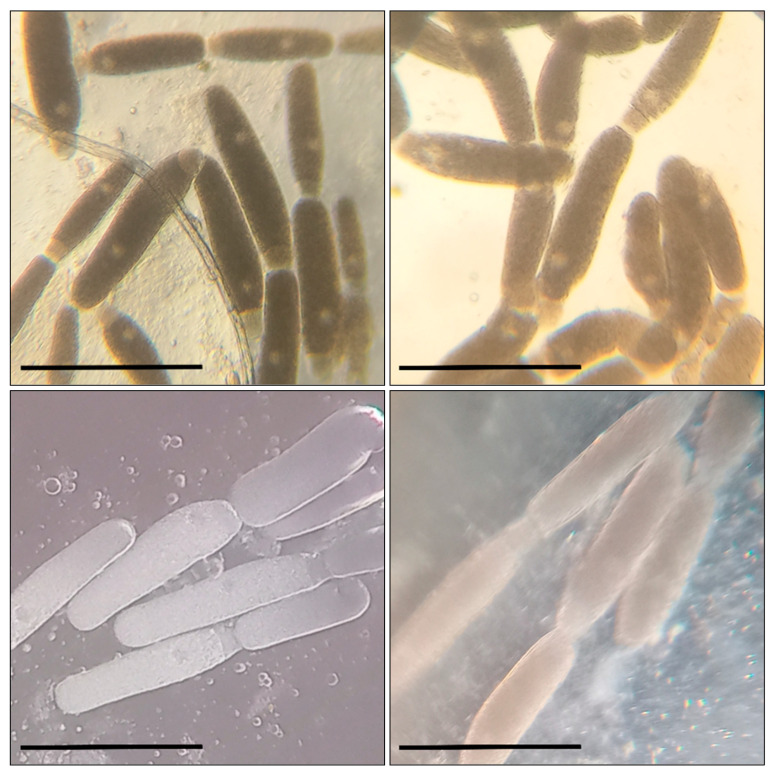
*Gregarina polymorpha* in the intestines of *T. molitor* larvae: bar—200 µm; the sizes of the gamonts and syzygies of gregarines, as well as the ratio of morphometric indices for the primite and satellite (the length and width of the protomerite and deutomerite, the location and size of the nucleus, etc.) correspond to the data indicated in the taxonomic literature.

**Figure 2:**
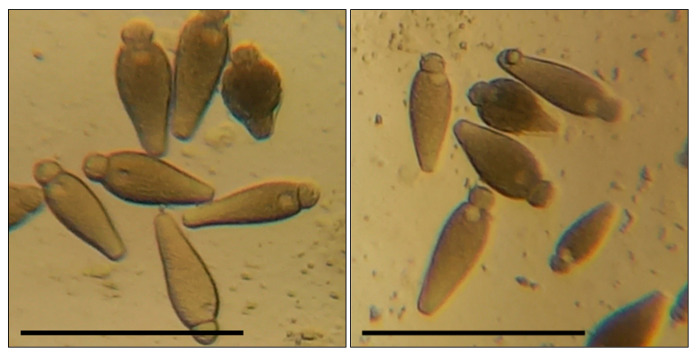
*Gregarina steini* in the intestines of *T. molitor* larvae: bar—100 µm; the body sizes of greragins and their morphometric indices correspond to the characteristics indicated in the work of Geus [56].

Before the larvae were transferred to a cup, each of them was weighed on scales with 1 mg accuracy. The mealworms were in the cups for 10 days, where their condition was checked regularly. On the fifth day of the experiment, we added 0.05 mL to support the effect of the tested compound on the insects’ development. After the experiment, the mealworms were weighed again to monitor changes in their body mass. Then, the intestines were removed from the larvae on a microscope slide; 12 transversal cuts were made on the intestines with a sharp blade at equal distance one from another. We carried out transverse sections of the middle intestine at an equal distance from each other in the segment between the stomach and the place of attachment of the Malpighian vessels to the intestine (the beginning of the hind intestine). Under a light microscope, we checked the temporary preparations of the intestines for gregarines, which, if found, were counted. In the mealworms used in the experiment, we found *G. polymorpha* (Figure 1) and *G. steini* (Figure 2), identified by microscopic measurements of their morphometric parameters and the guide by Geus [56]. The systematics were elucidated with regard to the new reports [25,26,32].

During the statistical analysis of the results, we used the standard methods of variance statistics (we estimated mean value, median, the first and third quartiles, and maximum and minimum values); significance of differences was estimated using ANOVA (analysis of variance). Differences between the values of the experimental groups were determined using the Tukey test (with consideration of Boniferroni’s correction), where the differences were considered significant at *p* < 0.05. Estimations were carried out in the Statistica 12.0 software package (Statsoft Inc., Tulsa, OK, USA, 2018).

## 3. Results

Some organic compounds were lethal to some mealworms (Table 1). The greatest morbidity was seen after exposure to cyclopentanol (33.3%), 2-naphthol (33.3%), trihydroxybenzoic acid (26.6%), and 3-aminobenzoic acid (20.0%). In the control group, we saw no dead larvae, though some formed pupae.

**Figure 3:**
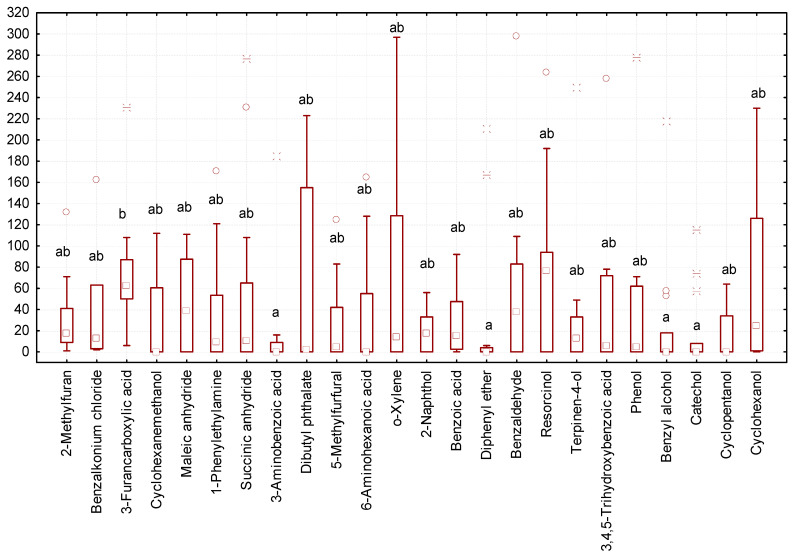
Number of *G. polymorpha* in the intestines of *T. molitor* at the end of the 10-day experiment (ordinate axis, spec.) in different variants of the experiment (N = 323): small square—median; lower and upper thresholds of the rectangle—the first and third quartiles, respectively; the lower and upper parts of the vertical line correspond to the minimum and maximum values of samplings; circles and stars ж—releases; different letters indicate values which reliably differed one from another within one line of the table according to the results of comparison using the Tukey test with Bonferroni correction.

In some variants of the experiment, we recorded a minimal number of gregarines (zero to three individuals of gregarines per larva, Figure 3). The lowest number of gregarines in the intestines was observed after exposure to diphenyl ether, benzyl alcohol, catechol, and 3-aminobenzoic acid. The highest number of gregarines was observed after exposure to resorcin and 3-furancarboxylic acid.

**Figure 4:**
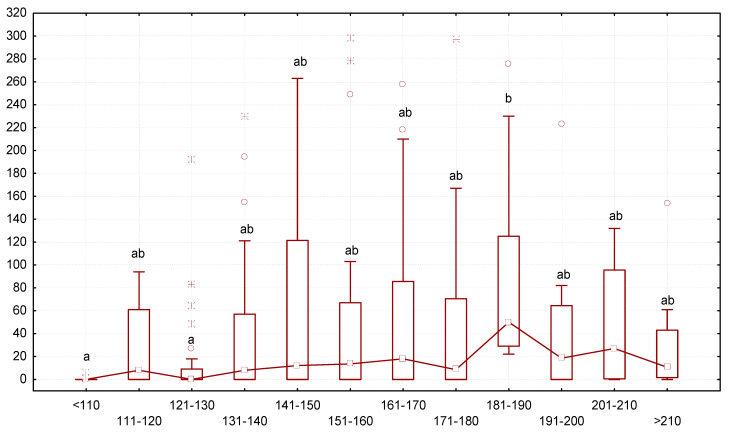
Dependence between the number of *G. polymorpha* (ordinate axis, number of specimens of gregarines per one *T. molitor*) and body mass of the insects (axis abscissa, mg of body mass): N = 323; legend—see Figure 3.

Analysis of the ratio of end mass and the number of gregarines in the larvae revealed that larvae with low body mass contained fewer gregarines, while larvae with greater body mass contained more gregarines. The intensity of gregarine infestation gradually increased starting from a body mass < 121–130 mg (Figure 4). The median number of gregarines reached the highest values in specimens with a body mass of 181–190 mg, measuring 52 gregarines per animal. If the body mass of the mealworms increased further, the number of gregarines had a downward tendency.

**Figure 5:**
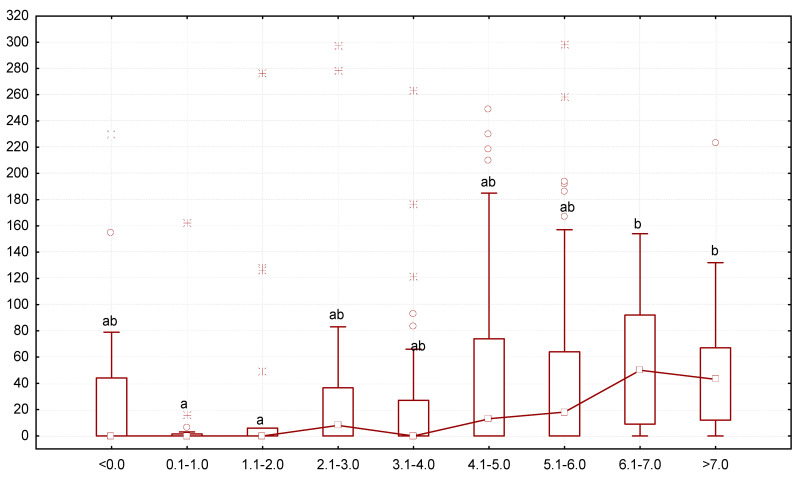
Dependence between the number of *G. polymorpha* (ordinate axis, number of gregarine specimens in one host) and change in body mass of the *T. molitor* larvae (abscissa axis, mg/day): N = 323; legend—see Figure 3.

The most active growth was noted for mealworms that contained greater numbers of *G. polymorpha*: the larvae that grew 0.1–1.0 or 1.1–2.0 mg/day or had been losing body mass (<0.0 mg/day) had the lowest number of gregarines, but as the body mass increased, so did the infection (Figure 5). The number of gregarines per mealworm was the highest in specimens growing at the rate of 6.1–7.0 mg/day (median was 50 gregarine specimens per one larva).

**Figure 6:**
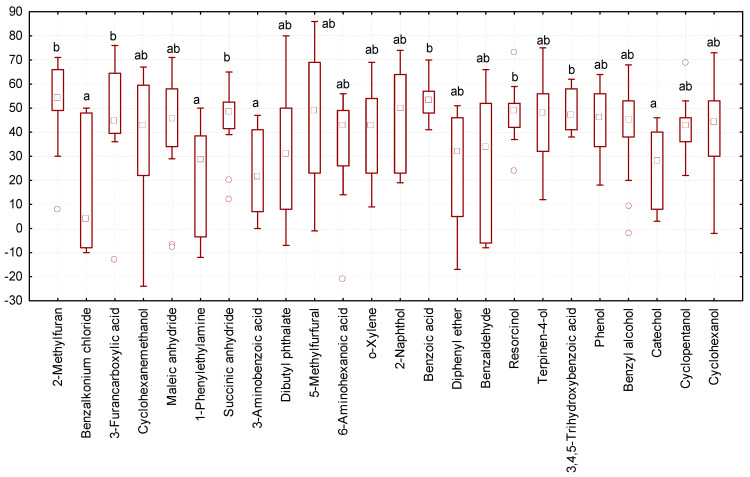
Changes in the body weight of *Tenebrio molitor* larvae at the end of a 10-day experiment (ordinate axis, mg) under the influence of various organic substances: N = 323; legend—see Figure 3.

We observed the maximum increase in body weight of larvae (median equal to 45–55 mg of body weight gain over the course of a 10-day experiment) when exposed to 3-furancarboxylic acid, 2-methylfuran, succinic anhydride, benzoic acid, resorcinol, and 3,4,5-trihydroxybenzoic acid (Figure 6). *Tenebrio molitor* larvae had a significantly lower increase in body weight (median 4–29 mg) in the experimental variants where they were exposed to benzalkonium chloride, 1-phenylethylamine, 3-aminobenzoic acid, and catechol.

## 4. Discussion

**Effects of the studied compounds on the mealworms.** Subjecting the beetles’ larvae to close contact with the organic compounds caused some to die. Because the compounds were able to affect the insects through their respiratory ways, enter the organisms with the swallowed substrate, and exert toxic properties, high mortality and a decline in the number of gregarines were expected to be a consequence of exposure to those compounds [57]. No larvae died in the normal conditions of the control group, where they grew without exposure to the compounds, though some formed pupae. In some variants of the experiment, the number of pupa was higher, although high mortality was not observed. Such cases could be explained by the age of the larvae that were ready to continue metamorphosis.

The highest mortality was recorded in the groups that had contact with cyclopentanol and 2-naphthol (33.0% dead in each group). Cyclopentanol is the main compound extracted from *Erica manipuliflora* Salisb, exerting strong insecticide properties [58], and 2-naphthol is a natural nematocide, extracted from *Actephila merrilliana* Chun [59]. Cyclopentanol is used as an intermediate product for colorings, solvent in perfumery, and pharmaceutics, while 2-naphthol is used to prepare colorings, pigments, perfumes, and drugs. Those compounds can affect the vitality of *T. molitor*.

**Effects of the studied compounds on gregarines.** By affecting the physical state of *T. molitor* larvae, the compounds also altered the life conditions in their bodies. In the intestines of the mealworms that were used in the experiment, we found *G. polymorpha* and *G. steini*. *Gregarina polymorpha* accounted for around 96% of the general population of gregarines. Invasion intensity with *G. polymorpha* ranged from 0 to 298 specimens per one individual of *T. molitor*. The intensity of gregarine infection varied across the variants of the experiment. The median number of gregarines was the highest in study variants with resorcin and 3-aminobenzoic acid, equaling 75 and 65 specimens of gregarines per host, respectively. In the concentrations we used, those compounds were unable to inhibit the state of gregarine trophozoites and reduce their quantity. The larvae that were exposed to diphenyl ether, 3-aminobenzoic acid, catechol, and benzyl alcohol had the lowest intensity of gregarine invasion. Diphenyl ether is a compound widely used in industry; its residues have been found in water, soil, air, and animal and human tissues [60]. This organic compound displayed antibacterial properties [61]. Similar effects were produced by 3-aminobenzoic acid, which is usually used for the synthesis of other compounds [62]; catechol, which is used mainly in the synthesis of insecticides [63]; and benzyl alcohol, which exhibited toxicity and insecticidal properties towards species of the Tenebrionidae family [54]. Our study demonstrated that the effects those compounds had on the larvae created an unfavorable environment for gregarines, while at the same time not increasing the mortality of the *T. molitor* significantly.

Despite the insecticidal properties of some substances, in the concentrations we specified, they did not significantly increase the mortality of larvae. However, the changes they caused in the host’s body affected the number of gregarines. This has the practical potential to breed gregarine-free populations of *T. molitor* with organic matter, which will be useful in further research on parasite systems.

In total, 6 out of 24 organic substances selected by us in one way or another destroyed the parasitic system “*Tenebrio molitor—Gregarina polymorpha*”, causing the death of the host or reducing the number of *Gregarina*. Each of these substances is used in certain industries, as part of medicines, insecticides, dyes, etc. Human activity increases the concentration of these substances in the environment. Urbanization, the growth of the local population, and the increase in the volume of production of goods lead to significant contamination with xenobiotics. Studies report the presence of xenobiotic compounds in surface waters around the world, including in the Arctic and Antarctica [50]. Despite low concentrations, xenobiotics are able to accumulate in the tissues of living organisms over time. In addition to living organisms encountering xenobiotics in natural ecosystems, they may also be exposed to them in laboratory populations. Food consumed by animals contains natural and synthetic chemicals that can affect physiological processes; insufficiently purified water may contain various pollutants [64]. It is important to consider this factor when studying living organisms and their interactions in different environments.

**Correlation between body mass and growth rates of the mealworms and gregarine-invasion intensity.** Our study revealed that the larvae with greater body mass had a high intensity of gregarine invasion. It gradually increased with the body mass of the host and reached the maximum in specimens that had a mass of 181–190 mg, with, on average, 52 gregarines per larva. With further increases in mass, infestation intensity gradually decreased. This may indicate an “optimum” body mass of the host for gregarines in the interval of 181–190 mg. Rates of body-mass increment positively correlated with the intensity of gregarine infection. This can be explained by the life cycle of *T. molitor*. By consuming food, the larva increases the intensity of invasion by gregarines, since infection with gregarines occurs through the ingestion of infectious oocysts together with food. After spending some time in the host’s body, gregarine gametocysts are released into the environment with feces. It can be assumed that larvae with a higher body weight and fewer gregarines were preparing for pupation: a certain time before it, they reduce their activity and stop consuming as much food, which leads to the fact that new gregarines do not enter the body.

This tendency remained in each variant of the study. Infestation with gregarines increased with the swallowing of infectious oocysts, and therefore we observed larger numbers of gregarines in the larvae that had to consume a greater amount of food with gregarine oocysts. A positive correlation between body mass and the number of gregarines was observed by Sumner [64]: he stated that infection by gregarines is necessary for the normal growth and long life of *T. molitor*. Later, research on the symbiosis of gregarines with *T. molitor* demonstrated that gregarines either affected the development of the host negatively or had no effect at all. Harry [65] found no significant difference between the end mass of infected *T. molitor* larvae and the end mass of non-infected larvae on an optimal diet. However, Harry [65] saw a substantial negative impact of gregarines on the larvae grown on a non-optimal diet. At the same time, some infected larvae grown in non-optimal feeding conditions lived much longer. The gregarine-infested orthopterans *Atractomorpha crenula* Fabricius, 1793 (Orthoptera, Pyrgomorphidae) consumed less food and, therefore, lost body mass [66]. The study of *Tribolium castaneum* Herbst, 1797 (Coleoptera, Tenebrionidae) revealed that gregarines hindered the growth of those insects, arrested their development, and increased their mortality [67]. Gregarines were seen to decrease the mass and length of *Tipula paludosa* Meigen, 1830 (Diptera, Tipulidae) [68]. Wolz et al. [69] demonstrated that gregarines additionally inhibited the state of insects that had already been subject to stress from insecticides. We observed none of those effects in our experiment. Gregarines consume nutrients from the host organism and can obstruct the midgut, causing health issues, but nonetheless, in our study we recorded a much larger number of gregarines than indicated in other sources [70]. The results suggest that even a high-intensity invasion does not harm the vitality or growth rates of *T. molitor* larvae.

The increase in the intensity of invasion by gregarines with an increase in the weight of the larva (and, accordingly, the amount of food it consumes) is natural, but a high load of gregarines must have consequences for the host, since gregarines tend to clog the midgut in case of a high-intensity invasion. But despite the fact that the intensity of *G. polymorpha* infection was much higher than indicated in other sources [70], we did not observe these complications. Gregarines are supposed to absorb nutrients from the host’s body, weakening it, but in our study it was shown that they do not interfere with the increase in the body weight of the larva. Although gregarines are considered parasites, in the case of *T. molitor* symbiosis with *G. polymorpha*, they do not cause visible damage to the host, even with high numbers.

## 5. Conclusions

In the concentrations we used, cyclopentanol and 2-naphthol inhibited the vitality of the mealworms, and 3-aminobenzoic acid, diphenyl ether, benzyl alcohol, and catechol decreased the invasion intensity by *G. polymorpha*. In the beetle larvae with lower body mass, the intensity of infestation with gregarines was lower, and the rates of the host’s body-mass increment correlated positively with a larger number of gregarines in their intestines. High-intensity loading with gregarines had no negative impact on the larvae.

## Figures and Tables

**Table 1:** Effects of organic compounds on survivability of mealworms (n = 15, duration of the experiment—10 days).

Compound	Number of Live Larvae	Number of Larvae That Turned into Pupae	Number of Larvae That Died
Cyclopentanol	9	1	5
2-Naphthol	9	1	5
3,4,5-Trihydroxybenzoic acid	11	0	4
3-Aminobenzoic acid	11	1	3
Benzalkonium chloride	7	6	2
Cyclohexanemethanol	8	5	2
Maleic anhydride	12	1	2
Phenol	13	0	2
Cyclohexanol	13	0	2
Resorcin	13	0	2
3-Furancarboxylic acid	9	5	1
1-Phenylethylamine	12	2	1
2-Methylfuran	13	1	1
Terpinen-4-ol	13	1	1
Dibutyl phthalate	13	1	1
Benzoic acid	14	0	1
Succinic anhydride	14	1	0
o-Xylene	14	1	0
Catechol	15	0	0
Diphenyl ether	15	0	0
Benzyl alcohol	15	0	0
5-Methyl furfural	15	0	0
6-Aminohexanoic acid	15	0	0
Benzaldehyde	15	0	0
Control group	20	10	0

## Data Availability

All data are either published with the manuscript or are available on request from the lead author.
